# The Adipocyte Acquires a Fibroblast-Like Transcriptional Signature in Response to a High Fat Diet

**DOI:** 10.1038/s41598-020-59284-w

**Published:** 2020-02-11

**Authors:** Jessica E. C. Jones, Nabil Rabhi, Joseph Orofino, Ramya Gamini, Valentina Perissi, Cecile Vernochet, Stephen R. Farmer

**Affiliations:** 1 Department of Biochemistry, Boston University School of Medicine, 72 East Concord Street, Boston, MA 02118 USA; 2 Internal Medicine Research Unit, Worldwide Research, Development and Medical, Pfizer Inc, 1 Portland Street, Cambridge, MA 02139 USA

**Keywords:** Transcriptomics, Diseases

## Abstract

Visceral white adipose tissue (vWAT) expands and undergoes extensive remodeling during diet-induced obesity. Much is known about the contribution of various stromal vascular cells to the remodeling process, but less is known of the changes that occur within the adipocyte as it becomes progressively dysfunctional. Here, we performed a transcriptome analysis of isolated vWAT adipocytes to assess global pathway changes occurring in response to a chronic high fat diet (HFD). The data demonstrate that the adipocyte responds to the HFD by adopting a fibroblast-like phenotype, characterized by enhanced expression of ECM, focal adhesion and cytoskeletal genes and suppression of many adipocyte programs most notably those associated with mitochondria. This study reveals that during obesity the adipocyte progressively becomes metabolically dysfunctional due to its acquisition of fibrogenic functions. We propose that mechano-responsive transcription factors such as MRTFA and SRF contribute to both upregulation of morphological genes as well as suppression of mitochondrial programs.

## Introduction

Obesity continues to be a global health issue with the prevalence rising in not only adults but also children^1^. Weight gain due to excess nutrition leads to expansion and remodeling of the adipose tissue^2^. Healthy adipocytes maintain a remarkably high degree of plasticity; quickly responding to whole body energy demands either through the release of fatty acids and glycerol, storage of excess calories as triglycerides and/or secretion of adipokines. The unique ability of the adipocyte to regulate its size through dynamic expansion or shrinkage requires continual remodeling of the tissue environment, specifically the extracellular matrix (ECM)^3–6^. The ECM is an important component of adipose tissue as it provides adipocytes with structural support and protection from mechanical stresses as well as serving as a reservoir of growth factors and cytokines^7–9^. Remodeling in healthy adipose tissue involves the continuous turnover of ECM proteins by a cycle of matrix deposition and degradation by enzymes such as metalloproteinases (MMPs)^10^. The maintenance of a pliable ECM is necessary to maintain the homeostasis of the tissue through the unrestricted expansion and contraction of adipocytes^3,4^. In addition to ECM turnover, angiogenesis within the fat pad keeps up with the expansion rate during healthy remodeling, ensuring that the tissue remains well oxygenated^11^. Maintenance of a flexible ECM is also necessary to allow for adipocyte progenitor cells to traverse to areas where new adipocytes are required^5^.

Unhealthy remodeling of adipose tissue due to over feeding involves enhanced deposition and decreased degradation or turnover of ECM, combined with an increase in crosslinking and stiffening of ECM fibers ultimately leading to adipocyte dysfunction, reduced adipogenic capacity and fibrosis with all contributing to whole body metabolic dysfunction, including type 2 diabetes and cardiovascular complications^12,13^. Indeed, deletion of collagen 6 (*col6)*, a component of the ECM that has enriched expression in adipose tissue, resulted in improved glucose tolerance and decreased liver triglycerides when challenged with an *ob/ob* background or a high fat diet^14^. Obesity-related remodeling also includes a change in various cell populations of the stromal vascular compartment. In lean individuals, adipose tissue is primarily composed of M2 macrophages, eosinophils, Tregs and ILCs that suppress inflammation. Whereas obesity is accompanied by an infiltration of B cells and various T cells (i.e. NK and Th1 cells) and polarization of M1 macrophages as well as a reduction in Tregs and ILCs leading to inflammation and associated insulin resistance. Secretion of proinflammatory cytokines also lead to expansion of profibrotic cells (ECM producing cells such as myofibroblasts) in addition to the inflammatory immune cells^15^.

In contrast to our extensive understanding of the many changes occurring in the stromal cell compartment of adipose tissue with obesity, less is known about the adipocyte-specific processes leading to an unhealthy adipocyte. The goal of this study was to evaluate the transcriptional response to HFD in isolated adipocytes. Our data demonstrate that adipocytes respond to a HFD by adopting a more fibroblast-like phenotype characterized by enhanced expression of ECM, cell adhesion and cytoskeletal genes along with suppression of adipocyte programs most importantly mitochondrial-related and lipolytic genes. Eventually, with prolonged high fat diet the transcriptional signature of the adipocyte is extensively altered from that of a healthy functional fat cell. We discuss the potential role of the morphological changes and associated transcriptional regulators in not only upregulating fibroblast-like genes but also in suppressing normal adipocyte functions.

## Methods

### Animal studies

C57BL/6J male mice were purchased from Jackson Laboratories (stock number 000664) and fed either a high fat diet (HFD) containing 60% kcal from fat (D12492; Research Diets) or control chow diet (D12450B or D12450K; Research Diets) starting from 6 weeks of age for 8, 20 or 34 weeks. Mice were maintained on a standard 12 hr light/dark cycle with ad libitum access to food and water. Bodyweights were measured prior to study and at the end of the study under fed conditions. On the final day of each study timepoint, mice were fasted for 6 hours (4:00–10:00am) by the removal of food but with free access to water. Mice were euthanized using carbon dioxide asphyxiation followed by cervical dislocation. All harvested tissues were weighed, and then either wrapped in foil and immediately snap-frozen in liquid nitrogen (LN_2_) or fixed in 10% neutral-buffered formalin for histological analysis. Subcutaneous adipose tissue was harvested from the inguinal region by carefully pulling the skin back from the flanks to reveal the triangular fad pads. Perigonadal adipose tissue, also known as epididymal adipose tissue, was harvested from the region surrounding the testes. With both adipose depots, careful attention was made to exclude lymph nodes during dissection At the completion of the study, tissues were stored at −80 °C.

All experimental protocols performed on animals were in accordance with regulations and established guidelines and were reviewed and approved by Institutional Animal Care and Use Committee (IACUC) at Pfizer Inc, Cambridge, MA.

### Adipose tissue fractionation

Dissociation media was prepared by adding 2 grams of bovine serum albumin (BSA) to 100 mL of 4.5 g/L glucose DMEM media (Invitrogen) and warmed in a 37 °C water bath to dissolve. Adipose tissue was dissected at the termination of the study. The fat pad was rinsed with PBS before being gently minced in dissociation media with scissors and a razor blade. The minced tissue was then digested by transferring to a 50 mL falcon tube containing 25 mL of 1 mg/mL type I collagenase (Worthington) dissolved in dissociation media and placed in a 37 °C water bath with gentle shaking for approximately 40 minutes. The dispersed tissue was then passed through a 100 µm mesh filter and centrifuged at 200xg for 10 minutes. The floating adipocytes were carefully removed and added to a tube containing approximately 5 volumes of trizol (Qiagen) and immediately snap frozen in a bath of LN_2_. The pelleted SVF was resuspended in erythrocyte lysing buffer (155 mM NH_4_Cl, 10 mM KHCO_3_, 1 mM EDTA), incubated at room temperature for 3 minutes and then centrifuged at 200xg for 10 minutes. Pelleted SVF was resuspended in 2 mL trizol (Qiagen) and immediately snap frozen in a bath of LN_2_. Cell fractions were stored at −80 °C.

### Body composition analysis

After 34 weeks of HFD, the relative total body fat mass and lean mass of mice was assessed by MRI scanning with an EchoMRI 700 instrument.

### Plasma collection and analysis of circulating plasma parameters

Whole blood was collected by cardiac puncture using a 26 G needle immediately following euthanasia by carbon dioxide and cervical dislocation. Whole blood was added to K_2_EDTA-containing collection tubes, gently inverted to mix and stored on ice before being centrifuged at 13,800xg for 10 minutes at 4 °C. Plasma was carefully removed and transferred to clean, pre-chilled microcentrifuge tubes on ice and analyzed immediately or stored at −80 °C for analysis at a later time. Glucose, ALT, AST, cholesterol, NEFA, glycerol, triglycerides and lactate were measured using a Siemens Advia 1800 Chemistry Analyzer (Siemens). Insulin was measured using Stellux Chemi Rodent Insulin ELISA kits (Alpco). Leptin was measured using Luminex magnetic bead assay kit (Thermofisher) according to manufacturer instructions.

### Histology and staining

Perigonadal adipose and liver tissues were processed with a standard paraffin-embedding protocol after fixation in 10% neutral-buffered formalin (Fisher Scientific), cut into 5 µm sections and mounted onto slides (Charles River). H&E staining was done by Charles River laboratories using their in-house protocols. Elastin staining was performed using an Elastica van Gieson staining kit (Millipore) according to manufacturer instructions. Picrosirius red staining was performed as previously described^16,17^. Bright field images for all stained tissue sections were captured with an Axio scan Z1 imager (Zeiss) with a 20x/0.8 objective. Digital zoom was used to select regions of interest and zoomed regions were cropped.

### Immunofluorescence of paraffin-embedded tissue sections

5 µm slices of paraffin-embedded perigonadal adipose tissue were mounted onto slides, deparaffinized and rehydrated before performing antigen retrieval. A Decloaking Chamber NxGen (Biocare Medical) was used for antigen retrieval according to manufacturer instructions. Briefly, slides were submerged in citrate buffer (10 mM sodium citrate, 0.05% tween-20, pH 6.0) and incubated for 15 minutes at 90 °C in the decloaking chamber. The slides were then cooled by submerging in room temperature water for 5 minutes and then rinsed twice with cold 0.1% tween-20 in tris-buffered saline (TBS) for 5 minutes. After the slides were rinsed, they were transferred to blocking solution (10% normal goat serum, Thermofisher) supplemented with 0.3 M glycine and 0.1% tween-20 and incubated at room temperature for 1 hour. After blocking, slides were rinsed twice with 0.1% tween-20 in TBS at room temperature. Wax circles were then drawn around the tissues and primary antibodies that had been diluted in 0.1% tween-20 TBS + 5% BSA as in Table 1 were applied and incubated overnight at 4 °C. The next day, slides were washed three times with 0.1% tween-20 TBS at room temperature. Secondary antibodies were diluted in 0.1% tween-20 TBS + 5% BSA along with DAPI as in Table 1. Slides were incubated with secondary antibodies in the dark for 1 hour. Slides were then washed three times with 0.1% tween-20 TBS at room temperature in the dark. Coverslips were mounted using Prolong gold antifade (Thermofisher). Fluorescent images for all stained adipose tissue sections were captured with an Axio scan Z1 imager (Zeiss) at 20x magnification. All chemicals were purchased from Sigma Aldrich.

**Table 1:** Table of antibodies.

Target	Species	Source	Catalog #	Dilution
Adiponectin (C45B10)	Rabbit	CST	2789	1:1000
ATGL (30A4)	Rabbit	CST	2439	1:1000
HSL (D6W5S) XP	Rabbit	CST	18381	1:1000
Phospho-HSL (Ser563)	Rabbit	CST	4139	1:1000
MAGL	Rabbit	TF	PA5-27915	1:1000
Cyclophilin A	Rabbit	CST	2175	1:1000
Anti-F4/80 antibody [CI:A3-1]	Rat	Abcam	ab6640	1:200
α-Smooth Muscle Actin (1A4) Mouse mAb (IF Formulated)	Mouse	CST	48938	1:400
CHOP (L63F7)	Mouse	CST	2895	1:200
Perilipin-1 (D1D8) XP	Rabbit	CST	9349	1:300
Collagen 6	Rabbit	Abcam	ab6588	1:400
Cleaved Caspase-3 (Asp175) (5A1E)	Rabbit	CST	9664	1:1000
Alexa Fluor 647 Phalloidin	N/A	CST	8940	1:20
Hoescht 33342	N/A	TF	H3570	1:10,000
Anti-rabbit IgG (H + L), F(ab’)2 Fragment (Alexa Fluor 488 Conjugate)	Goat	CST	4412	1:1000
Anti-rabbit IgG (H + L), F(ab’)2 Fragment (Alexa Fluor 594 Conjugate)	Goat	CST	8889	1:1000
Anti-rabbit IgG (H + L), F(ab’)2 Fragment (Alexa Fluor 647 Conjugate)	Goat	CST	4414	1:1000
Anti-mouse IgG (H + L), F(ab’)2 Fragment (Alexa Fluor 647 Conjugate)	Goat	CST	4410	1:1000
Anti-mouse IgG (H + L), F(ab’)2 Fragment (Alexa Fluor 594 Conjugate)	Goat	CST	8890	1:1000
Anti-rat IgG (H + L) (Alexa Fluor 555 Conjugate)	Goat	CST	4417	1:1000
Anti-rabbit IgG, HRP-linked Antibody	Goat	CST	7074	1:10,000
Anti-mouse IgG, HRP-linked Antibody	Horse	CST	7076	1:10,000

Table of antibodies used for western blot and immunofluorescence.

CST = Cell Signaling Technologies. TF = ThermoFisher.

Fluorescent images for all stained adipose tissue sections were captured with an Axio scan Z1 imager (Zeiss) with a 20x/0.8 objective. All images were compared against background control slides containing secondary fluorophore-conjugated antibodies only. Histogram display adjustments were made for each fluorophore and were kept the same across samples. Digital zoom was used to select regions of interest and zoomed regions were cropped.

### Frozen tissue preparation

Frozen adipose tissue was powdered by gently hammering in foil on a metal pedestal partially submerged in LN_2_. Frozen pieces of tissue were hammered until pulverized to a fine, uniform powder and then transferred to a vial, pre-chilled in LN_2_. Powdered tissues were then returned to −80 °C for storage or prepared for further analysis.

### Protein isolation and western blot analysis

Total adipose tissue lysates were made by scooping approximately 200 mg powdered adipose tissue (prepared as described above) into a microcentrifuge tube on ice containing 400 µL RIPA buffer (ThermoFisher) supplemented with 1% triton X-100 (Sigma) and 1x protease and phosphatase inhibitor cocktail (Cell Signaling Technologies). After vortexing to ensure thorough mixing, samples were sonicated for 2 rounds of 3 seconds at 30 mHz with a brief pause in between rounds. Careful attention was paid to ensure the samples did not warm during sonication. After sonication, the samples were cleared by centrifugation at 18,800xg for 15 minutes at 4 °C. The supernatant was careful removed, avoiding the lipid cake at the surface as well as particulates at the bottom of the tube. The supernatant was transferred to a clean, pre-chilled microcentrifuge tube and cleared again as described above. After clearing, protein concentrations were determined by BCA assay (Pierce). For western blot analysis, equal amounts of protein from cleared tissue lysates were prepared with 4x Nupage LDS loading buffer (ThermoFisher) and 1x reducing agent (DTT; ThermoFisher) before being heated for 10 minutes at 70 °C. After a brief cooling period until no longer warm to the touch, the samples were loaded into 26 lane 4–20% Tris-Glycine midi gels (Biorad) and separated by SDS-PAGE according to manufacturer instructions (Biorad). After SDS-PAGE was complete, the proteins were then transferred to a pre-activated and transfer buffer equilibrated polyvinylidene difluoride (PVDF) membrane (Biorad) by wet tank transfer. The wet transfer conditions were 90 minutes at 85 V keeping the transfer chamber cold with an internal ice pack and surrounding the chamber with ice. Transfer buffer was 1x Tris/Glycine transfer buffer (Biorad) supplemented with 20% methanol (Fisher). Following transfer, PVDF membranes were blocked with 5% non-fat milk (Biorad) in tris-buffered saline supplemented with 0.2% tween-20 (TBS-T; 50 mM Tris-Cl, pH 7.5, 150 mM NaCl, 0.2% tween-20) for 1 hour at room temperature with gentle rocking. Membranes were then rinsed with TBS-T before being incubated overnight at 4 °C with gentle rocking. Primary antibodies prepared in 5% BSA/TBS-T with dilutions specified in Table 1. The following day, primary antibodies were removed and the membranes were washed 3 × 5 minutes with TBS-T buffer. After washing, membranes were incubated with appropriate HRP-linked secondary antibodies diluted in 5% non-fat milk in TBS-T for 1 hour at room temperature with gentle rocking. Membranes were then washed 3 × 5 minutes with TBS-T buffer. Proteins were detected using SuperSignal West Femto Maximum Sensitivity Substrate kit (ThermoFisher). Membranes were exposed and images were captured using a ChemiDoc XRS + system (Biorad) with careful monitoring of exposure time to avoid saturation of signal. All antibodies and dilutions used are listed in Table 1. Densitometry analysis was done using Image Lab 5.2.1 software (BioRad).

### RNA and cDNA preparation

Isolated adipocytes and stromal vascular cells snap frozen in trizol were thawed on ice and then transferred to Fastprep matrix D lysing tubes (Mp Biomedicals) for homogenization using a Qialyzer instrument (Qiagen). Cells were homogenized with 2 cycles of 2 minutes at 20 1/s frequency, rotating tubes within holders (outside to inside and vice-versa) between rounds. RNA was extracted from the tissue homogenates using the RNeasy Lipid Tissue Mini kit and the Qiagen user-developed protocol titled Purification of total RNA from fatty tissues using the RNeasy Lipid Tissue Mini Kit and MaXtract High Density (Qiagen). On-column DNA digest was performed for all samples according to manufacturer protocol (Qiagen). RNA was quantified using a Nanodrop 8000 spectrophotometer and quality was assessed using an Agilent 4100 Bioanalyzer (Agilent). cDNA was synthesized from RNA using High-capacity cDNA reverse transcription kits (Applied Biosystems).

### Gene expression

Real-time quantitative PCR (qPCR) was performed with a Quantstudio 7 flex instrument (Applied Biosystems) using Taqman Fast advanced mastermix (Applied Biosystems) and appropriate taqman probes for the genes of interest. Relative expression was normalized to housekeeping genes (Ppia and Tbp). All taqman probes were purchased from ThermoFisher and are listed in Table 2.

**Table 2:** Taqman probes used for qPCR.

Gene	Catalog #
*Acta2*	Mm00725412
*Col1a1*	Mm00801666
*Col4a1*	Mm01210125
*Col6a3*	Mm00711678
*Ctgf*	Mm01192933
*Fn1*	Mm01256744
*Inhba*	Mm00434339
*Itga5*	Mm00439797
*Mmp11*	Mm00485048
*Mmp2*	Mm00439498
*Mmp9*	Mm00442991
*Serpine*	Mm00435858
*Sparc*	Mm00486332
*Tgfb1*	Mm01178820
*Tgfbr1*	Mm00436964
*Tgfbr2*	Mm03024091
*Timp1*	Mm01341361
*Tnc*	Mm00495662

All probes were purchased from ThermoFisher.

### RNAseq library preparation

cDNA libraries for RNA-seq analysis were prepared from total RNA (isolated as described above) according to Illumina TruSeq stranded mRNA sample prep LS protocol. Pooled sample libraries were sequenced on Nextseq. 500 instrument with Nextseq. 500/550 High output kit, 150 cycles (Illumina).

### Gene expression profiling (read mapping, quantification and differential expression)

#### Stromal vascular fraction analysis

Raw sequencing reads were subjected to quality control analysis via FastQC^18^ and reports aggregated via MultiQC^19^. Reads were then trimmed based on a sliding window and presence of adapter sequences using Trimmomatic^20^ set to the following parameters: ILLUMINACLIP::2:30:10 LEADING:3 TRAILING:3 SLIDINGWINDOW:4:15 MINLEN:50. Salmon^21^ was used to quantify the expression of transcripts to a reference transcriptome containing protein coding and long non-coding RNAs from the gencode M10 annotation before aggregation to the gene level. Only genes with a raw count of greater than 5 and present in at least half of the replicates were used for downstream analysis. Normalization and differential expression were performed using DESeq2^22^ set to default parameters. All raw sequencing data has been deposited to Gene Expression Omnibus record number GSE138242.

#### Isolated adipocytes analysis

The raw RNA-seq fastq files were processed using the count-based differential expression analysis best practice protocol^23^ to quantify for gene expression. Accordingly, sequencing reads were aligned to the mouse genome mm10 using the STAR aligner^24^. FeatureCounts was used to count the number of reads unambiguously overlapping each gene, where each gene was considered to be the union of its exons^25^ with GENCODE annotation (vM2 for mouse). Sample normalization factors were computed using the EdgeR TMM method^26^. Unless otherwise stated, all counts per million (CPM) and log2CPM values are computed using these normalization factors. We converted the gene symbols to Entrez IDs using the annotations in the mygene.info package^27^. Of the 38924 genes, 9690 gene symbols did not have an associated Entrez ID and were discarded from this study. Only genes with gene-expression data above log2CPM of 2.0 were considered expressed, filtering the rest, resulting in a gene list of 11600 informative genes. The resulting genes with Entrez IDs correspond to the set of ‘background or detected genes’ consisting of 11600 genes. The biological replicates for each condition were pooled for differential expression analysis. Genes with a q-value of < 0.05 were considered significantly differentially expressed. All raw sequencing data has been deposited to Gene Expression Omnibus record number GSE142187.

### Transcriptional pathway analysis of RNAseq data

Differentially expressed genes were selected based on padj < 0.05. GSEA pathway enrichment analysis was conducted on differentially expressed gene lists that were ranked by logFC^28,29^. Lists of differentially expressed genes were also analyzed for enriched GO terms^30,31^ using the online EnrichR tool^32,33^ and Revigo^34^. Differentially regulated genes associated with mitochondria were identified by comparing gene lists to the MitoCarta^35,36^.

### Statistical analysis

Data were analyzed using GraphPad Prism 7 software (GraphPad) and are presented as mean ± standard error of mean (SEM). Group comparisons were analyzed using either two-tailed unpaired student t test or a two-way ANOVA followed by multiple comparisons correction method stated in figure legend. Differences were deemed statistically significant with p < 0.05.

## Results

### Diet-induced obesity leads to progressive metabolic impairment over 34 weeks

**Figure 1:**
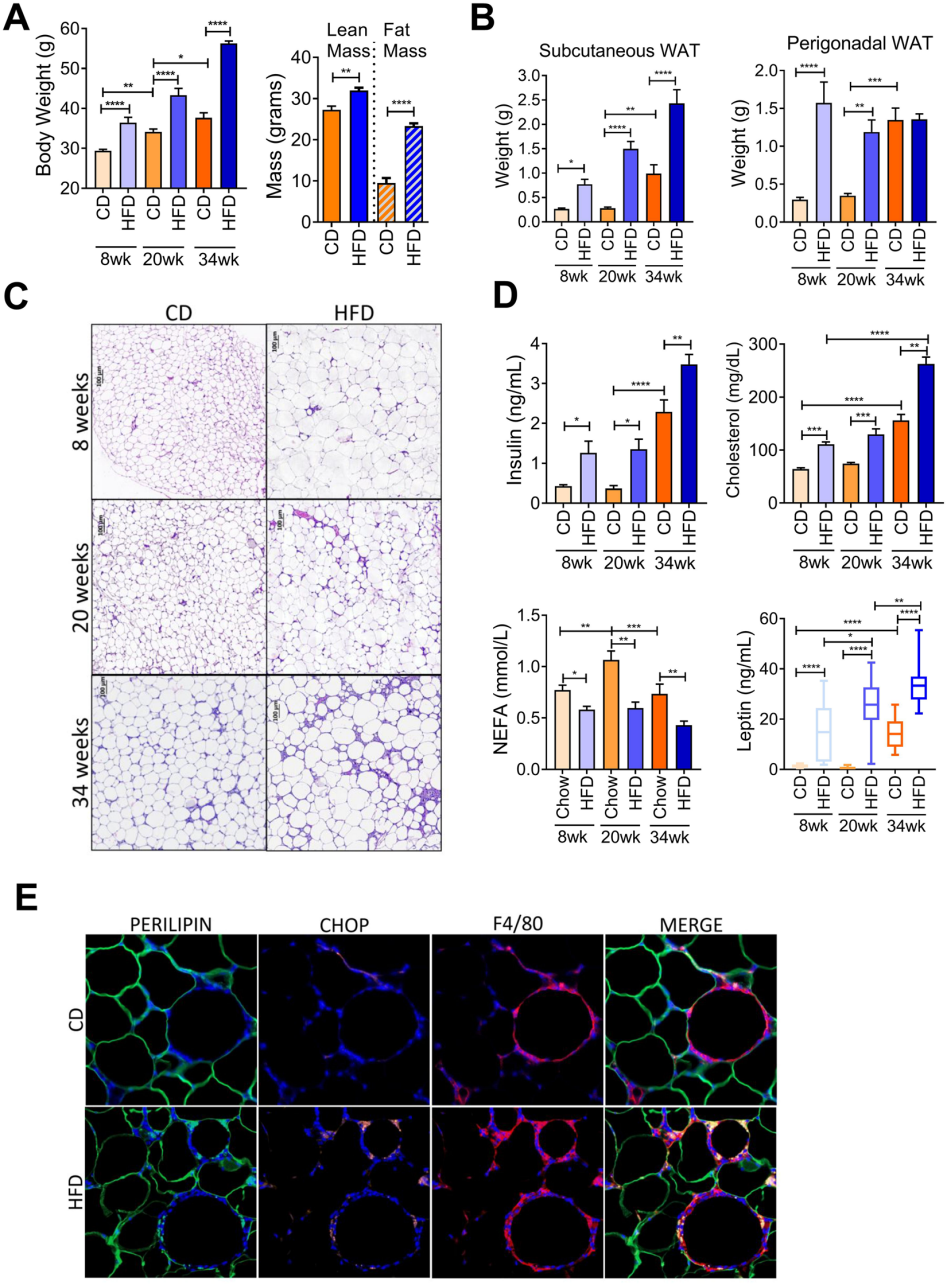
High fat diet feeding over the course of 34 weeks leads to increased bodyweight, fat mass and metabolic impairment. (**A**) High fat diet (HFD) feeding over the course of 34 weeks resulted in significantly increased bodyweight compared to chow diet (CD). At 34 weeks, body composition was measured using EchoMRI to determine relative fat and lean mass. (**B**) Subcutaneous adipose and perigonadal adipose tissue weights of chow diet (CD) or high fat diet (HFD) fed mice over a course of 34 weeks. (**C**) Hematoxylin and eosin staining (H&E) of cross-section of perigonadal adipose tissue from chow diet (CD) or high fat diet (HFD) fed mice after 8, 20 and 34 weeks. (**D**) Circulating plasma analytes were measured after a 6 hour fast. N = 5–8 mice per group. (**E**) Immunofluorescent staining of paraffin-embedded PGWAT from chow diet (CD) or high fat diet (HFD) fed mice for 34 weeks. Perilipin (green), CHOP (orange), F4/80 (red) and nuclei (blue). Representative image from 3–4 mice. Means ± SEM. Comparisons between groups and statistical analysis done with two-way ANOVA controlling for false discovery rate (FDR < 0.05) with Two-stage step-up method of Benjamini, Krieger and Yekutieli or unpaired t-tests with two-tailed p-values. *p < 0.05, **p < 0.01, ***p < 0.001, ****p < 0.0001.

To begin to evaluate adipose tissue remodeling in the context of weight gain and progression of obesity, we fed C57/Bl6J mice either chow diet (CD) or 60% high fat diet (HFD) over a time course of 8, 20 and 34 weeks starting from 6 weeks of age. Most HFD diets studies have analyzed the effect of the diet on overall metabolism for relatively short periods (8–20 weeks). Here, we were interested in determining what effect a chronic diet (34 weeks) would have on the mice compared to their normal weight gain with age. As anticipated, mice fed either CD or HFD gained weight as time progressed and mice fed HFD gained significantly more weight compared to CD groups at all time points (Fig. 1A). Body composition analysis revealed that after 34 weeks of HFD, total lean mass was modestly increased, and total fat mass increased approximately 2-fold compared to CD group (Fig. 1A). In agreement with weight gain, both subcutaneous and perigonadal adipose depots expanded in mass. Subcutaneous fat pads were larger and continued to increase in weight with time compared to perigonadal fat pads that had reached a plateau from the 8 weeks of HFD timepoint (Fig. 1B). Examination of perigonadal adipose tissue cross sections stained with Hematoxylin and Eosin (H&E) revealed that HFD led to larger adipocytes but also enhanced staining intensity surrounding the adipocytes compared to CD at all times (Fig. 1C). Next to gain insight into the metabolic health of the mice, we measured fasting insulin, cholesterol, NEFA and leptin in the plasma. Fasting insulin and circulating cholesterol were elevated with HFD at all times (Fig. 1D) compared to CD. Circulating NEFAs were transiently increased in the CD with time and were decreased in the HFD group at all times (Fig. 1D). Circulating leptin rose in a similar pattern to the expansion of subcutaneous fat pads (Fig. 1B,D) in response to HFD compared to CD. Liver weight and circulating enzymes associated with liver damage, alanine aminotransferase (ALT) and aspartate transferase (AST), were significantly elevated after 34 weeks of HFD (Supplementary Fig. 1A,C) compared to CD. Progressive lipid accumulation in the liver in the HFD groups was also evident by H&E staining of tissue sections (Supplementary Fig. 1C). Thus, collectively confirming that HFD-fed mice were becoming increasingly metabolically impaired over time. Moreover, the age-associated changes in mice fed chow seem to recapitulate some of the HFD phenotypes at earlier time points. Immunofluorescent staining of the perigonadal adipose tissue sections with a macrophage marker, F4/80, confirmed that the clustering around adipocytes was populated with macrophages (Fig. 1E). Assessment of CHOP expression, a marker of ER stress^37^, by immunofluorescent staining after 34 weeks of HFD revealed elevated expression within the crown-like structures surrounding the adipocytes (Fig. 1E). Furthermore, CHOP expression colocalized more frequently with F4/80 expression in the HFD-fed group (Fig. 1E) compared to CD group.

### Adipocytes upregulate expression of extracellular matrix genes in response to high fat diet

**Figure 2:**
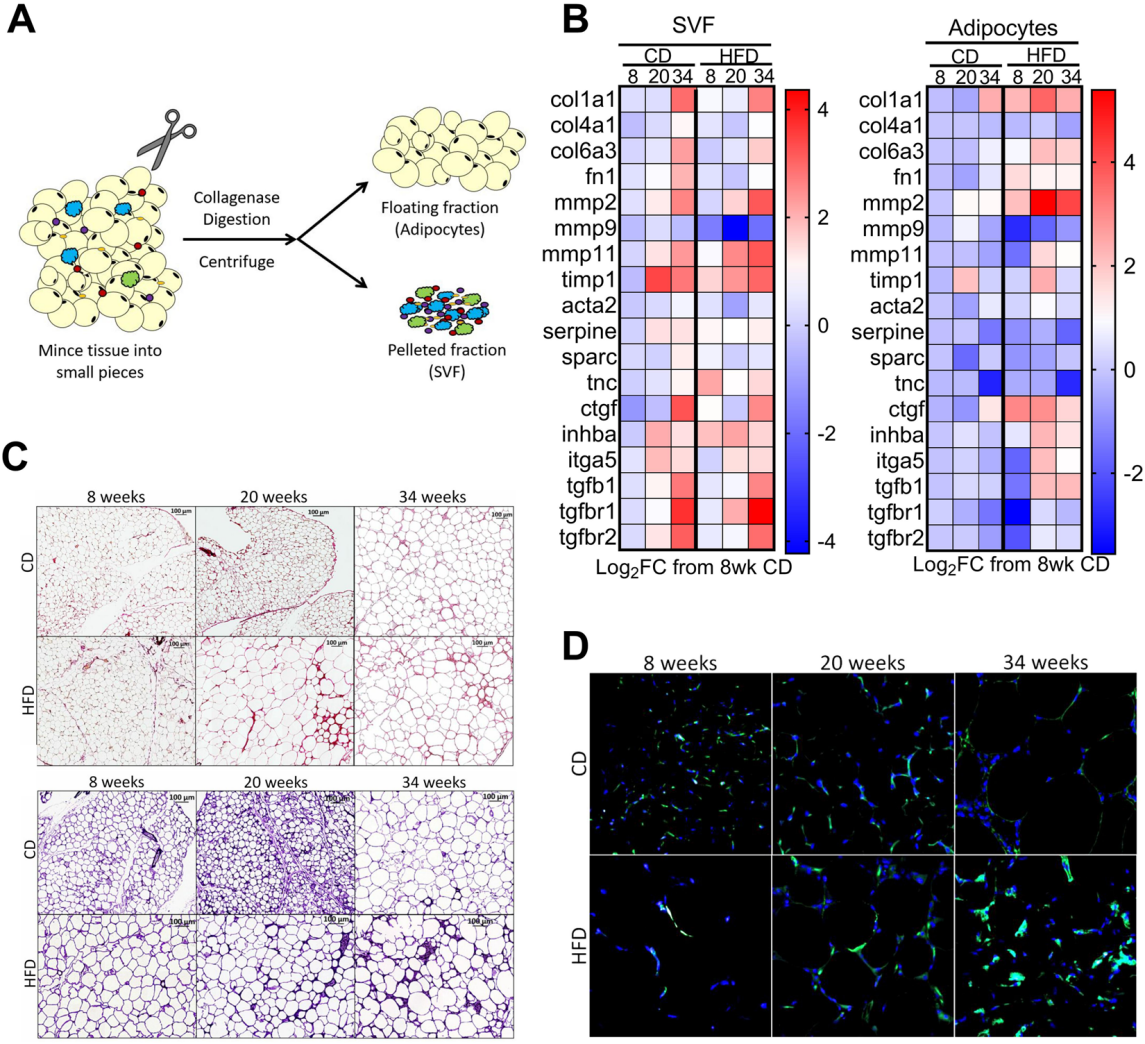
High fat diet feeding over 34 weeks leads to enhanced expression of extracellular matrix and remodeling components. (**A**) Illustration of fractionation procedure of perigonadal adipose tissue. (**B**) Gene expression of ECM remodeling and fibrosis markers in SVF cells (left) and isolated adipocytes (right) as measured by qPCR. Data are represented as log2 fold change (Log2FC) from 8 week chow diet (CD) group. Heat map colors represent median values. N = 5–8 mice per group. (**C**) Picrosirius red staining (detecting collagen) of cross-section of perigonadal adipose tissue from chow diet (CD) or high fat diet (HFD) fed mice after 8, 20 and 34 weeks (top panel). Elastica van Gieson staining (detecting elastin) of cross-section of perigonadal adipose tissue from chow diet (CD) or high fat diet (HFD) fed mice after 8, 20 and 34 weeks (bottom panel). All images taken with 20x objective with 8% digital zoom. Scale bar represents 100 µm. (**D**) Immunofluorescent analysis of α-SMA (green) expression in PGWAT from chow diet (CD) or high fat diet (HFD) fed mice after 8, 20 and 34 weeks. Nuclei are colored blue. Representative image from 3–4 mice.

To specifically analyze the 2 main cell fractions of adipose tissue, adipocyte (AF) and stromal vascular fractions (SVF), the perigonadal depot (PGWAT) was digested with collagenase as briefly outlined in Fig. 2A. Gene expression was assessed in both PGWAT fractions by qPCR for a selected set of genes associated with extracellular matrix (ECM) and tissue remodeling (Fig. 2B). The gene expression of these remodeling markers in the SVF cells appeared to be influenced greater by age than by HFD as shown by similar fold increases and pattern of gene expression between the 2 diet groups (Fig. 2B, left panel). Expression of collagens (*col1a1*, *col4a1*, *col6a3*) and fibronectin (*fn1*) were similarly upregulated in SVF from CD and HFD groups at 34 weeks (Fig. 2B, left panel). Tissue remodeling enzymes *mmp2*, *mmp9*, *mmp11* and *timp1* expression was also similar between the groups (Fig. 2B, left panel). TGF-β1 (*tgfb1*), its receptors (*tgfbr1*, *tgfbr2*) as well as expression of its target genes *(itga5*, *acta2*, *ctgf*, *serpine*) were all similarly expressed in SVF cells in response to HFD and time (Fig. 2B, left panel). One marker that was more strongly upregulated in response to high fat compared to CD in SVF was *tnc*. Tnc is a pro-fibrotic glycoprotein that has been shown to contribute to tissue remodeling and fibrosis through its interaction with toll-like receptor 4 (TLR4), a receptor associated with pro-inflammatory responses^38–41^. In contrast to SVF cells, AF cells responded strongly to high fat stimulus compared to CD (Fig. 2B, right panel). Expression of collagens (*col1a1* and *col6a3*), fibronectin (*fn1*) were strongly upregulated in the AF from HFD PGWAT starting at 8 weeks and progressing to 34 weeks compared to CD adipocytes (Fig. 2B, right panel). Expression of matrix metalloproteinases, *mmp2* and *mmp11*, were elevated in response to HFD, and a tissue inhibitor of metalloproteinases, *timp1*, displayed a similar expression pattern for diet and time (Fig. 2B, left panel). *Tgfb1*, *inhba*, *itga5* and *ctgf* were all strongly upregulated in response to HFD at 20 and 34 weeks compared to CD (Fig. 2B, left panel). In contrast to SVF, *tnc* was strongly downregulated in the AF by 34 weeks independent of high fat stimulus (Fig. 2B, left panel). Given the increased expression of multiple ECM and tissue remodeling genes, we next assessed PGWAT tissue cross sections with histological staining for collagen (Picro Sirius Red) and elastin (Elastica van Gieson). Pericellular collagen staining was particularly apparent after 20 and 34 weeks HFD compared to CD (Fig. 2C, top panel), and became localized around the larger adipocytes at 34 weeks in the PGWAT of both CD and HFD mice (Fig. 2C, top panel). Elastin staining was similar to that of collagen (Fig. 2C, bottom panel). Interestingly, both collagen and elastin staining displayed the highest intensity in the areas of the tissue in which there was obvious SVF remodeling, the macrophage-enriched crown-like structures surrounding the dying adipocytes^42^. It has been shown that adipose tissue macrophages associate with elastin fibers and that macrophages are the predominant producers of MMP-12, a degrading enzyme for elastin, suggesting that macrophages play an active role in elastin remodeling^43^. Curiously, there appeared to be a transient increase in overall elastin staining in the 20 week CD group (Fig. 2C, bottom panel). This is interesting because it occurred before the tissue went on to nearly triple in weight by the 34 week timepoint, perhaps suggesting a time of remodeling in response to an unknown stimulus. Alpha smooth muscle actin (α-SMA) immunofluorescent staining of perigonadal tissue sections revealed an interesting pattern in CD mice (Fig. 2D). At 8 weeks, the expression of α-SMA appeared to be confined to cells distributed throughout the tissue, which then became more ordered around adipocytes by 20 weeks (Fig. 2D). Intriguingly at 34 weeks, α-SMA expression was delicately outlining the adipocytes in addition to concentrating between them in areas of higher nuclei density (Fig. 2D). In comparison, α-SMA positive cells in the 34 week HFD group appeared to have concentrated localization (Fig. 2D). These cells could be myofibroblasts and/or blood vessel cells.

### Adipocytes exert a robust transcriptional response to high fat diet

To take an in-depth look at the transcriptional changes occurring globally within the different fractions of the PGWAT, we conducted RNA-seq analysis on the isolated SVF and adipocytes of CD and HFD fed mice at 8, 20 and 34 weeks. After 8 weeks, the number of differentially expressed genes (DEGs) in the SVF in response to HFD was limited to a few hundred (Supplemental Fig. S2A,B). Interestingly after 20 weeks of HFD the number of DEGs substantially increased in the SVF before a subsequent overall decrease at 34 weeks (Supplemental Fig. S2A,B). Conversely, the adipocytes appeared to respond more strongly to HFD. After 8 weeks of HFD, the number of DEGs in adipocytes was approximately 5500 genes (Supplemental Fig. S2A,B). After 20 weeks, the number of DEGs in adipocytes reached a maximum, as was seen in the SVF, before modestly decreasing in total number by 34 weeks (Supplemental Fig. S2A,B).

**Figure 3:**
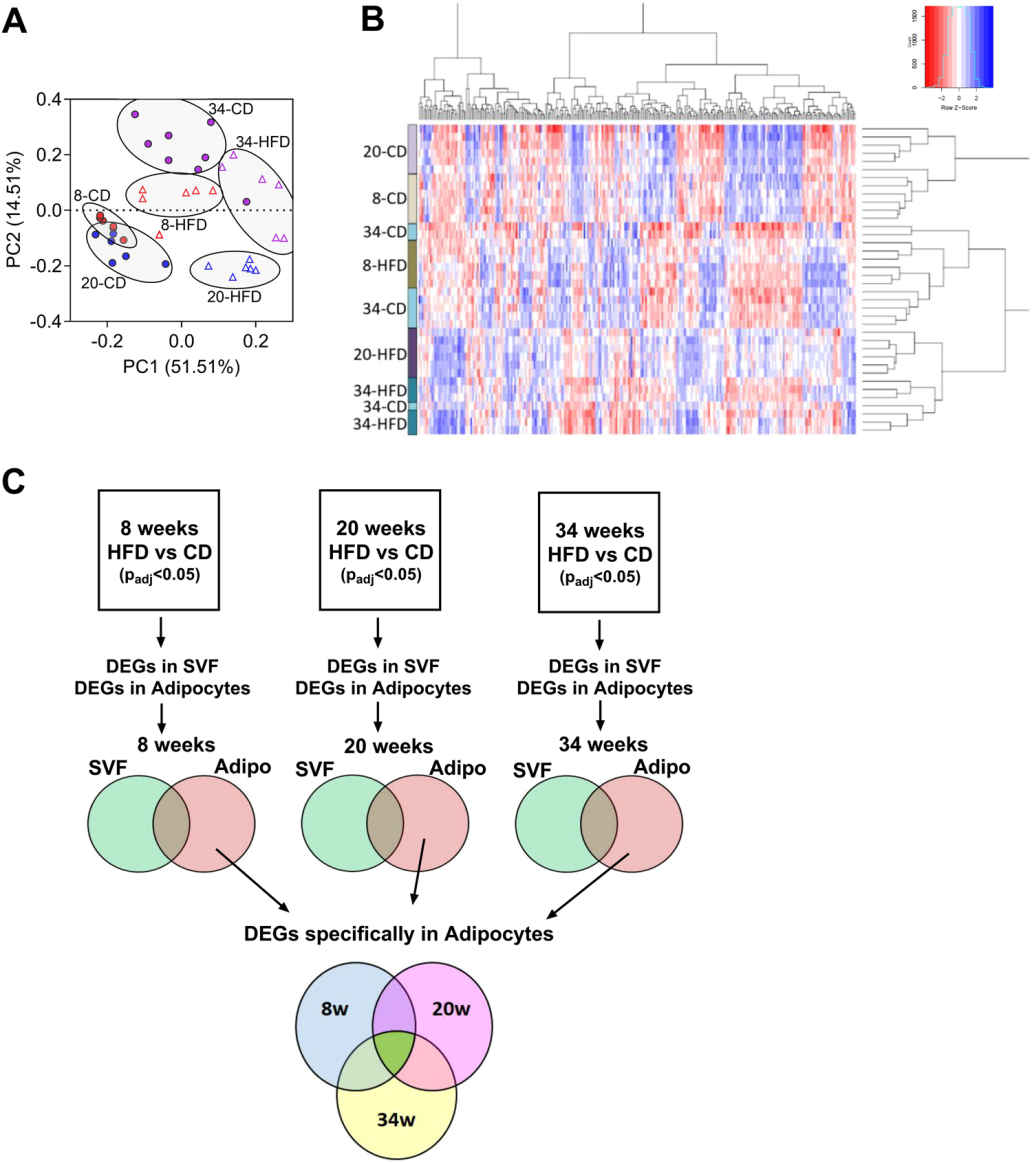
Adipocytes exert a robust transcriptional response to high fat diet. (**A**) Principal component analysis of differentially expressed genes from isolated perigonadal adipocytes from chow diet (CD) or high fat diet (HFD) groups after 8, 20 or 34 weeks. Chow diet (CD) groups are closed circles. High fat diet (HFD) groups are open triangles. 8 week timepoint is red, 20 week timepoint is blue and 34 week timepoint is purple. Each point represents one sample. 6–8 mice per group. (**B**) Hierarchical clustering heatmap of isolated adipocytes. (**C**) Overview of workflow for GO term pathway analysis. Analysis overview for selecting differentially expressed genes (DEGs) that are specifically up or downregulated in adipocyte fraction.

Principal component analysis of the adipocytes revealed clustering of the diet groups and timepoints. This analysis facilitates a high level overview of similarities and differences between the samples and groups in relation to how they cluster^44^ (Fig. 3A). 8 and 20 week CD samples clustered closely together and the groups partially overlapped, suggesting similarities between them (Fig. 3A). 8, 20 and 34 week HFD samples clustered together within their respective groups and were near each other however there was no overlap between any of the groups (Fig. 3A). Thus suggesting that high fat was eliciting global transcriptional changes leading to high level differences between the groups at earlier timepoints compared to CD. Intriguingly, the 34 week CD samples clustered nearer the 8 and 20 week HFD groups than the 8 or 20 week CD groups (Fig. 3A). This is consistent with the data in Fig. 1, which showed that changes in metabolic parameters following 34 weeks of chow diet were similar to those resulting from 8 or 20 weeks of HFD. Hierarchical clustering analysis highlighted the directional changes occurring globally within the different diet groups and timepoints (Fig. 3B). To perform a more detailed transcriptional profiling of the adipocytes, we took a reductionist approach to correct for any carryover of the SVF into the adipocyte fraction. An overview of the workflow is detailed in (Fig. 3C). After selection of DEGs (both up and downregulated) from the SVF and adipocytes in response to HFD compared to CD based on padj < 0.05, the lists were overlaid at each timepoint to reveal genes that were differentially expressed only in the SVF (Fig. 3C, first row of Venn diagrams, green color), in the adipocyte fraction (Fig. 3C, first row of Venn diagrams, red color) or in both fractions (Fig. 3C, first row of Venn diagrams, brown color) at each of the times. Next, we took the lists of DEGs that were specifically changing in the adipocyte fraction from each of the timepoints (Fig. 3C, first row of Venn diagrams, orange color) and overlaid them using a final Venn diagram (Fig. 3C, lower Venn diagram with 3 circles). This final comparison allowed us to identify DEGs that were differentially expressed across all timepoints (Fig. 3C, lower Venn diagram, bright green color) or specifically at each timepoint (Fig. 3C, lower Venn diagram, blue, pink and yellow colors). Once these lists were made, GO term enrichment analysis was performed to determine the dominant pathways that were changing in the adipocytes in response to HFD at 8, 20 and 34 weeks as well as what pathways were in common over all timepoints. All DEGs for both SVF and adipocyte fraction are listed in Supplemental File 1.

### Upregulation of ECM and remodeling-related genes and pathways in high fat diet adipocytes

**Figure 4:**
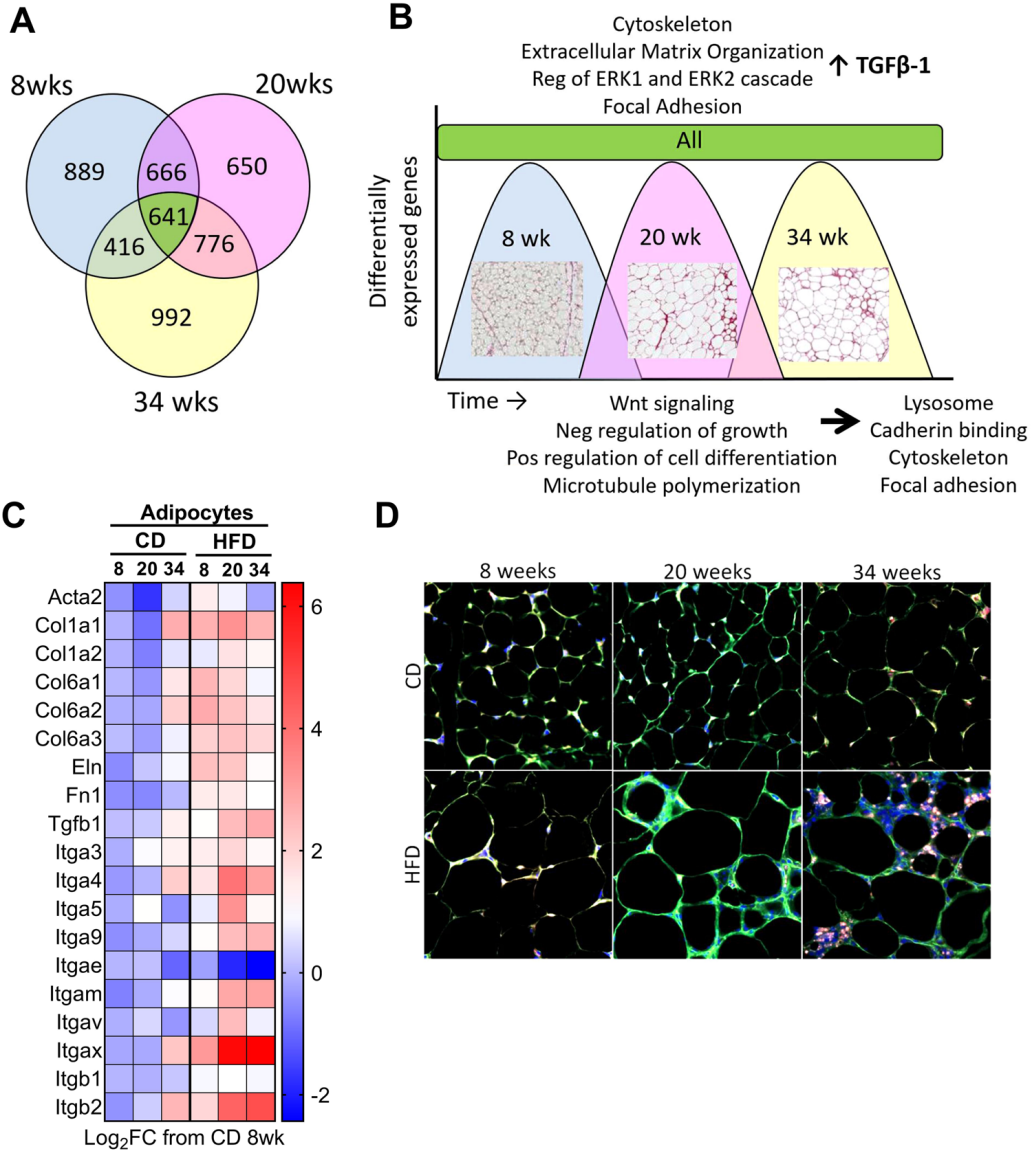
Adipocytes upregulate extracellular matrix associated genes in response to high fat diet. (**A**) Venn diagram of upregulated differentially expressed genes in adipocytes. (**B**) GO term analysis of upregulated pathways at each timepoint or across all timepoints. Pos = positive. Neg = negative. Representative picrosirius red staining images are overlaid for each timepoint. (**C**) ECM and remodeling genes extracted from RNAseq analysis (TPM values) expressed as Log_2_FC from 8 week CD. Heat map colors represent median values. N = 5–8 mice per group. (**D**) Immunofluorescent staining of paraffin-embedded PGWAT tissue sections from chow diet (CD) or high fat diet (HFD) fed mice after 8, 20 and 34 week for collagen 6 (green), F-actin (stained with phalloidin-red) and nuclei (blue). Representative image from 3–4 mice.

641 DEGs were maintained upregulated over all timepoints in response to high fat stimulus in isolated adipocytes (Fig. 4A, green color). GO term analysis revealed enrichment of pathways related to extracellular matrix organization, cytoskeleton, focal adhesion and others (Fig. 4B, green color). GO terms and associated genes for Fig. 4B are listed in [Supplemental Fig. 3 and Supplemental File 2]. Notably, pro-fibrotic cytokine TGF-β1 (Tgfb1) expression was found to be upregulated in adipocytes at all times (Supplemental File 2). 8 weeks of HFD lead to upregulation of 889 genes which were surprisingly not significantly associated with any GO terms (Fig. 4A, blue color). By 20 weeks of HFD, adipocytes significantly upregulated 650 genes associated with Wnt signaling, negative regulation of cell growth, positive regulation of cell differentiation and microtubule polymerization (Fig. 4A,B, pink color). Genes associated with these GO terms, including other TGF-β ligands *tgfb2* and *tgfb3*, are listed in Supplemental Fig. 3 and Supplemental File 2. Thirty four weeks of HFD lead to 992 genes uniquely upregulated (Fig. 4A, yellow color) which equated to the enrichment of GO terms associated predominantly with RNA metabolic process, lysosome, cadherin binding, cytoskeleton, focal adhesion and others (Fig. 4B, yellow color). 34 week upregulated GO terms and associated genes are listed in Supplemental Fig. 3 and Supplemental File 2.

Upregulation of a subset of ECM and remodeling genes in the isolated adipocytes with HFD was evident after calculating the Log_2_FC from RNAseq TPM values (Fig. 4C). Collagens (*col1a1, col6a3*), elastin (*eln*), fibronectin (*fn1*), TGFB family members (*tgfb1*), and integrins (*itga5*) were all upregulated in adipocytes in response to a HFD. Next, we examined collagen 6 (COL6), an enriched collagen in adipose tissue^14^, and f-actin expression (using phalloidin^45^) by immunofluorescent staining of PGWAT tissue sections (Fig. 4D). We found that both proteins were more prominent with HFD, particularly at the later timepoints (Fig. 4D). Interestingly, by 34 weeks HFD the phalloidin staining was more punctate than in previous timepoints (Fig. 4D). COL6 signal appeared to decrease in CD mice over the time, suggesting active degradation and remodeling was occurring in response to the expanding adipocytes.

### Downregulation of mitochondrial genes and GO terms in high fat diet adipocytes

**Figure 5:**
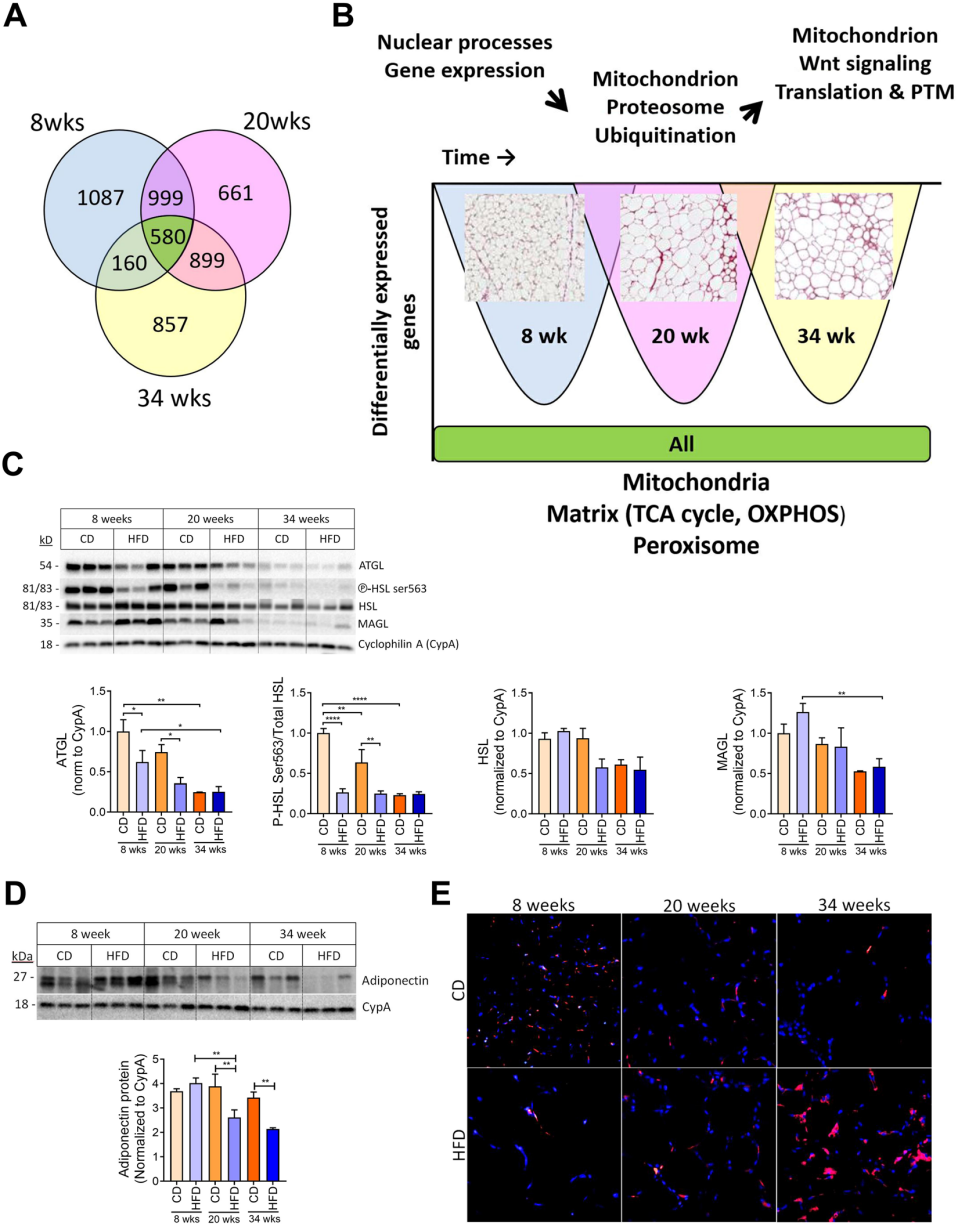
Adipocytes downregulate mitochondria associated genes and pathways in response to high fat diet. (**A**) Venn diagram of downregulated differentially expressed genes in adipocytes. (**B**) GO term analysis of downregulated pathways at each timepoint or across all timepoints. Representative picrosirius red staining images are overlaid for each timepoint. (**C**) Western blot analysis of total PGWAT lysates for lipolysis mediators. Data are normalized to housekeeping protein, cyclophilin A (CypA). (**D**) Western blot analysis of total PGWAT lysates for adiponectin. Data are normalized to housekeeping protein, cyclophilin A (CypA). (**E**) Immunofluorescent staining of paraffin-embedded PGWAT tissue sections from chow diet (CD) or high fat diet (HFD) fed mice after 8, 20 and 34 weeks for cleaved caspase-3 (red) and nuclei (blue). Representative image from 3–4 mice. Means ± SEM. Comparisons between groups and statistical analysis done with two-way ANOVA controlling for false discovery rate (FDR < 0.05) with Two-stage step-up method of Benjamini, Krieger and Yekutieli or unpaired t-tests with two-tailed p-values. *p < 0.05, **p < 0.01, ****p < 0.0001.

Adipocytes had a similar number of downregulated genes at each timepoint as those being upregulated (Fig. 5A). Notably, 580 genes were downregulated at all times and of these genes the top GO term was mitochondrion (Fig. 5A,B, green color, Supplemental Fig. S4). After 8 weeks of HFD, 1087 genes were downregulated specifically at this time (Fig. 5A, blue color). These genes enriched for GO terms primarily related to RNA processing and transcription (Figs. 5B and S4). After 20 weeks of HFD 661 genes were downregulated, out of which 72 were associated to the top enriched GO term mitochondria (Fig. 5, pink color and Supplemental Fig. S4). Other downregulated GO terms were associated with the peroxisome and ubiquitination. After 34 weeks of HFD, 857 were found to be specifically downregulated and GO terms associated with the mitochondrion became more prevalent including mitochondrion inner membrane, mitochondrial translation and mitochondrion (Fig. 5, yellow color and Supplemental Fig. S4). Genes associated with downregulated GO terms at all time points are listed in Supplemental File 2. Given the known concept of mitochondrial dysfunction occurring during obesity, we next examined proteins important for adipocyte function (lipolysis) and health (adiponectin)^46^. Western blot analysis of total PGWAT lysates showed a decrease in lipolytic enzymes, ATGL and phosphorylated ser563 HSL, a PKA site that leads to activation of HSL^47^, after 8 weeks of HFD (Fig. 5C). Total HSL and MAGL expression declined at the later 20 and 34 week times (Fig. 5C). Adiponectin also decreased after 20 weeks of HFD and continued to decrease during the remainder of the 34 weeks (Fig. 5D). Immunofluorescence of cleaved caspase-3 (a marker of autophagy^48^) appeared dispersed and relatively small in area in 8 week CD, becoming more scarce with time however larger in area. 8 week HFD treatment resembled the 34 week CD. HFD treatment lead to increasing cleaved caspase-3 activity during the 34 week period suggesting increased apoptosis with obesity (Fig. 5E).

## Discussion

It is well documented that white adipose tissues particularly visceral depots undergo an extensive remodeling in response to high fat diet (HFD), which includes deposition of ECM and infiltration of proinflammatory immune cells. With time, HFD causes inflammation and fibrosis that results in dysfunctional adipose depots and disruption of metabolic homeostasis. Much is known about the contribution of the stromal cells (immune, vascular, fibroblasts etc) to the unhealthy remodeling, but little is known about the response or contribution of the adipocytes. In the present study, we performed a RNAseq analysis of isolated adipocytes to assess global pathway changes occurring in response to HFD. Our data reveal that adipocytes respond dramatically to the diet by enhancing expression of genes coding for ECM, focal adhesions and cytoskeleton, which is maintained throughout the entire 34 weeks of the diet. In fact, the transcriptional signature of the resultant pathological adipocytes resembles that expressed prior to their adipogenesis, i.e. more similar to fibroblastic-like cells. This is intriguing and somewhat controversial however there is precedence for adipocytes to revert to preadipocytes or fibroblast-like cells in other tissues undergoing remodeling in response to stress. Dermal adipocytes have the flexibility to transition to myofibroblasts^49^. In scleroderma, the skin undergoes extensive remodeling resulting in fibrosis of the tissue. Scleroderma myofibroblasts have been shown to originate from subdermal adipocytes^49^. The data further demonstrate that after 20 weeks of HFD, genes associated with Wnt signaling are significantly upregulated. Wnt signaling has been shown to be implicated in dermal fibrosis^50^. Furthermore, surgical-induced injury to subcutaneous fat pads in rats led to the dedifferentiation of mature adipocytes to cells with a fibroblast morphology^51^. Mammary adipocytes have been shown to dedifferentiate into PDGFRα + cells during lactation^52^. PDGFRα is an accepted marker of precursor cells^53^.

It is well accepted that adipose tissue becomes progressively fibrotic during diet-induced obesity due to the extensive deposition of ECM around adipocytes as well as blood vessels. In other organs, fibrosis is characterized by proliferation and differentiation of perivascular progenitors into myofibroblasts that reorganize and contract the ECM fibers providing mechanical stimulations within the tissues^54–57^. Recent studies suggest that similar mechanisms operate in adipose tissue and contribute to its unhealthy state^58–62^. Other studies have suggested that the adipocyte plays a central role in adipose fibrosis by expressing ECM genes in response to hypoxia^63,64^. Our data also support a role for the adipocyte but GO terms enrichment analysis of HFD upregulated genes suggests that pathways responsive to TGF-β are a principal regulator of adipocyte-specific production of ECM, focal adhesions and cytoskeleton. This does not exclude a role for hypoxia since its stimulation of collagen type VI and endotrophin could lead to release of TGF-β from immune cells^65,66^.

TGF-β regulates gene expression through several overlapping signaling/ transcription factor pathways including SMADs, JNK, ERKs and MRTFA/SRF. Our earlier studies have provided data implicating MRTFA as a factor contributing to diet-induced metabolic disruption of adipose tissue by regulating the fate of perivascular progenitors to favor fibrogenesis over adipogenesis^60,67^. The healthier phenotype of HFD fed MRTFA-deficient mice compared to controls could also be due to its absence in mature adipocytes thereby preventing conversion of healthy adipocytes to dysfunctional fibroblast-like cells. Similarly, absence of SMAD3 signaling protects mice from diet-induced obesity and insulin resistance in part by enhancing production of beige adipocytes in white adipose tissue^68^. SMAD3 might also have detrimental effects on obese adipocytes. Strategies to inhibit TGF-β signaling (SMADs and MRTFA) might limit the negative consequences of obesity by directly retaining the metabolic functions of adipocytes.

The dramatic acquisition of a more fibroblast-like program of gene expression will have a direct impact on the morphological integrity of the adipocyte. An important feature of a metabolically healthy adipocyte is its ability to expand and contract in response to lipid storage and release. The unique morphology of the fat cell facilitates these changes in volume due to formation of a cortical cytoskeleton surrounding the lipid droplet rather than an extensive network of filaments throughout the cytoplasm as in the case of fibroblastic cells. Such a filamentous network most likely forms in the obese adipocyte due not only to actin production but also expression of focal adhesions that anchor to ECM externally and cytoskeleton internally providing mechanical tension across the cell. A stiff ECM surrounding them but also an actin-cytoskeleton internally acting to constrain expansion of the adipocyte with accumulation of lipid. Early studies demonstrated the need to downregulate the cytoskeleton and ECM during adipogenesis in order to facilitate formation of a unilocular fat cell^69,70^.

Besides providing the adipocyte with its plasticity, the cortical cytoskeleton also contributes to the normal metabolic functions of the adipocyte such as glucose uptake^71^. It is also conceivable that adipocyte tubulins play a role in regulating mitochondrial activity^72,73^. Consequently, a dramatic rearrangement of actin and tubulin cytoskeleton could have a detrimental effect on normal adipocyte metabolic functions. In fact, our data show that in addition to upregulation of ECM and cytoskeletal genes, there is a corresponding down-regulation of other genes. Most notably, 580 genes were downregulated at all times and of these genes the top GO term was mitochondrion. Other programs down-regulated included those also playing central roles in normal healthy adipocyte functions including lipolysis and adiponectin production. Down-regulation of these programs could be related to changes in overall morphology but are more likely due to suppression of transcriptional factors and/or chromatin rearrangements by the same signaling pathways that upregulated ECM and cytoskeletal genes. As discussed earlier, MRTFA/MKL1 along with SRF respond to extracellular signals via the polymerization state of actin to enhance expression of the morphological genes^74–76^. These same factors however antagonize the activity of PPARγ^67,77–79^, which is a master regulator of genes controlling normal adipocyte functions including glucose uptake and mitochondrial activity. Therapies being developed for tissue fibrosis that target signaling pathways regulating TGFβ-associated transcription factors might be effective in preventing the decline in adipocyte-specific functions during obesity.

## Supplementary information

Supplementary Dataset 1.
