# Supplementary material for: The Adipocyte Acquires a Fibroblast-Like Transcriptional Signature in Response to a High Fat Diet
Source: Sci Rep. 2020 Feb 11;10:2380. doi: 10.1038/s41598-020-59284-w (PMC7012923; doi:10.1038/s41598-020-59284-w)
Supplement: Supplementary file 1 — Supplementary Dataset 1. [file 41598_2020_59284_MOESM1_ESM.pdf]

The Adipocyte Acquires a Fibroblast-Like Transcriptional Signature in Response to a High Fat Diet

Jessica E.C Jones[1,2], Nabil Rabhi[1], Joseph Orofino[1], Ramya Gamini[2], Valentina Perissi[1], Cecile Vernochet[2] and Stephen R. Farmer[1*]

Supplemental Figures

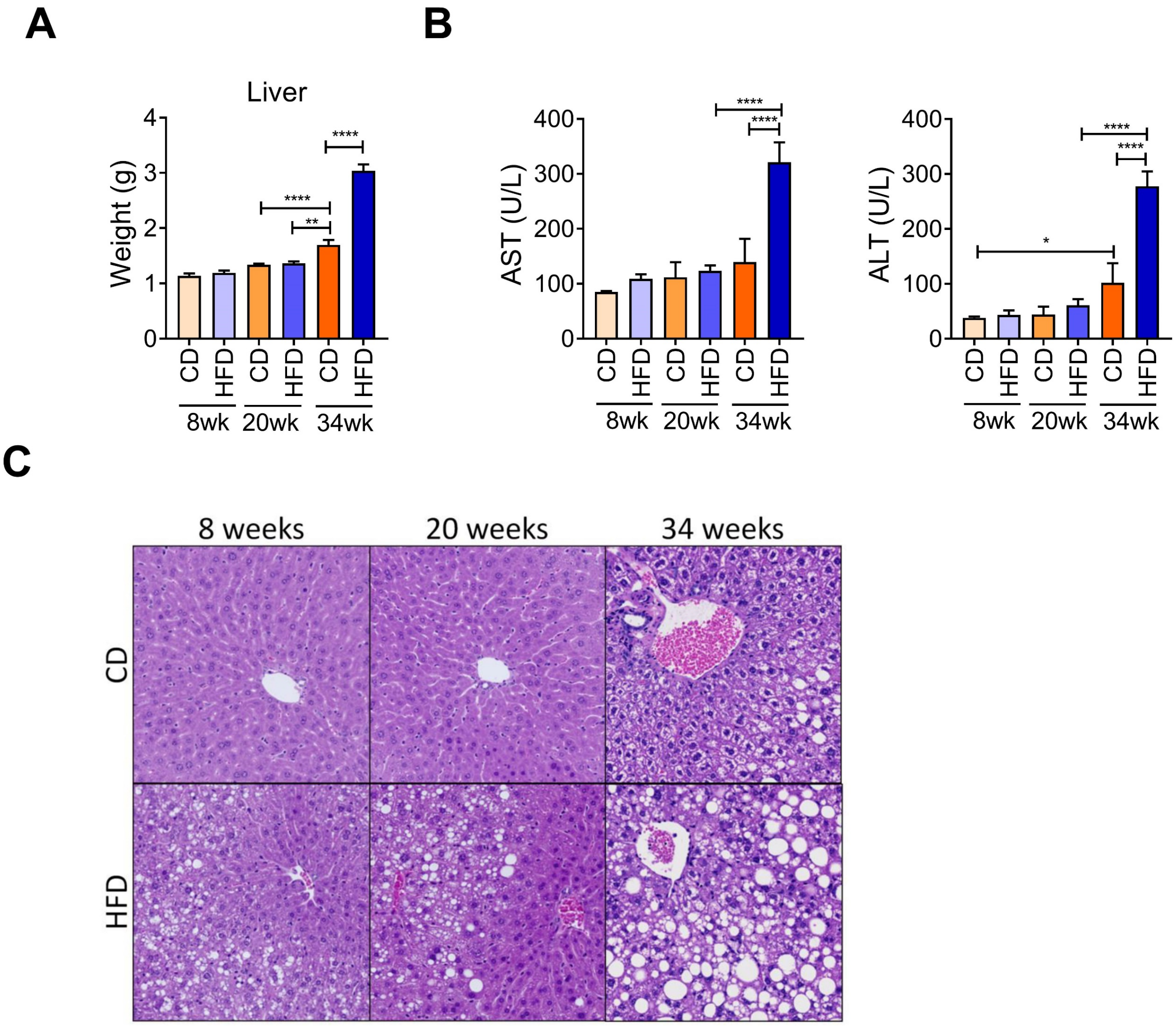

Supplemental Figure 1: High fat diet leads to increased lipid accumulation and release of liver enzymes.

A) Liver tissue weights of chow diet (CD) or high fat diet (HFD) fed mice over a course of 34 weeks. B) Circulating AST and ALT after a 6 hour fast and study completion. C) Hematoxylin and eosin staining (H&E) of cross-section of liver tissue from chow diet (CD) or high fat diet (HFD) fed mice after 8, 20 and 34 weeks. N=5-8 mice per group. Comparisons between groups and statistical analysis done with two-way ANOVA controlling for false discovery rate (FDR<0.05) with Two-stage step-up method of Benjamini, Krieger and Yekutieli or unpaired t-tests with two-tailed p-values. *p<0.05, **p<0.01, ****p<0.0001.

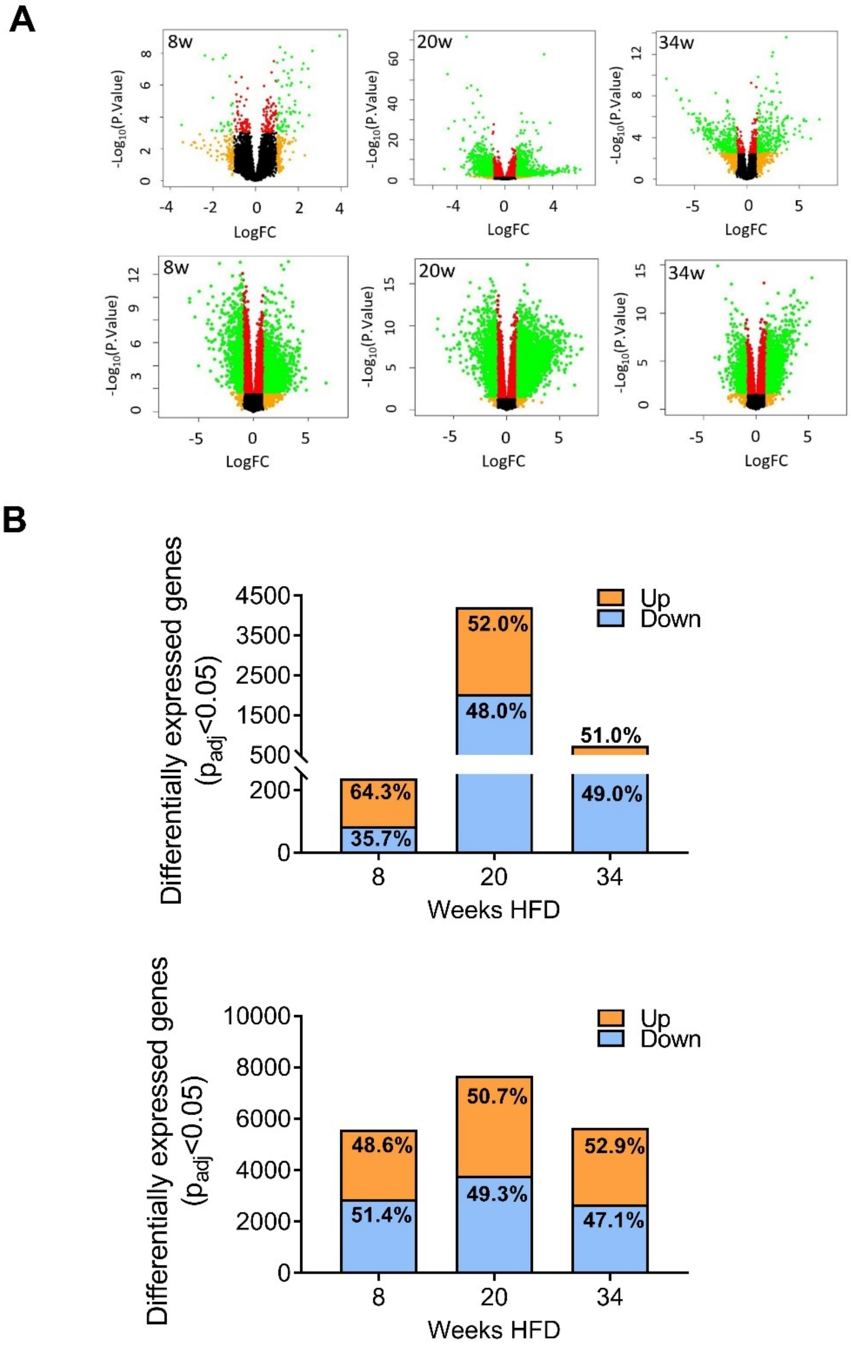

Supplemental Figure 2: Differential gene expression analysis of fractionated PGWAT.

A) Volcano plots of differentially expressed genes in high fat diet compared to chow diet in SVF (top row) or isolated adipocytes (bottom row) from the perigonadal adipose depot after 8 weeks (8w), 20 weeks (20w) and 34 weeks (34w). Red = genes with adjusted p<0.05, orange = genes with logFC >1, green = genes with adjusted p<0.05 and logFC > 1, black = genes that did not fulfill any of these criteria. B) Differentially expressed genes and associated percentage of total genes for SVF (top) and adipocyte fraction (bottom) over the course of the study.

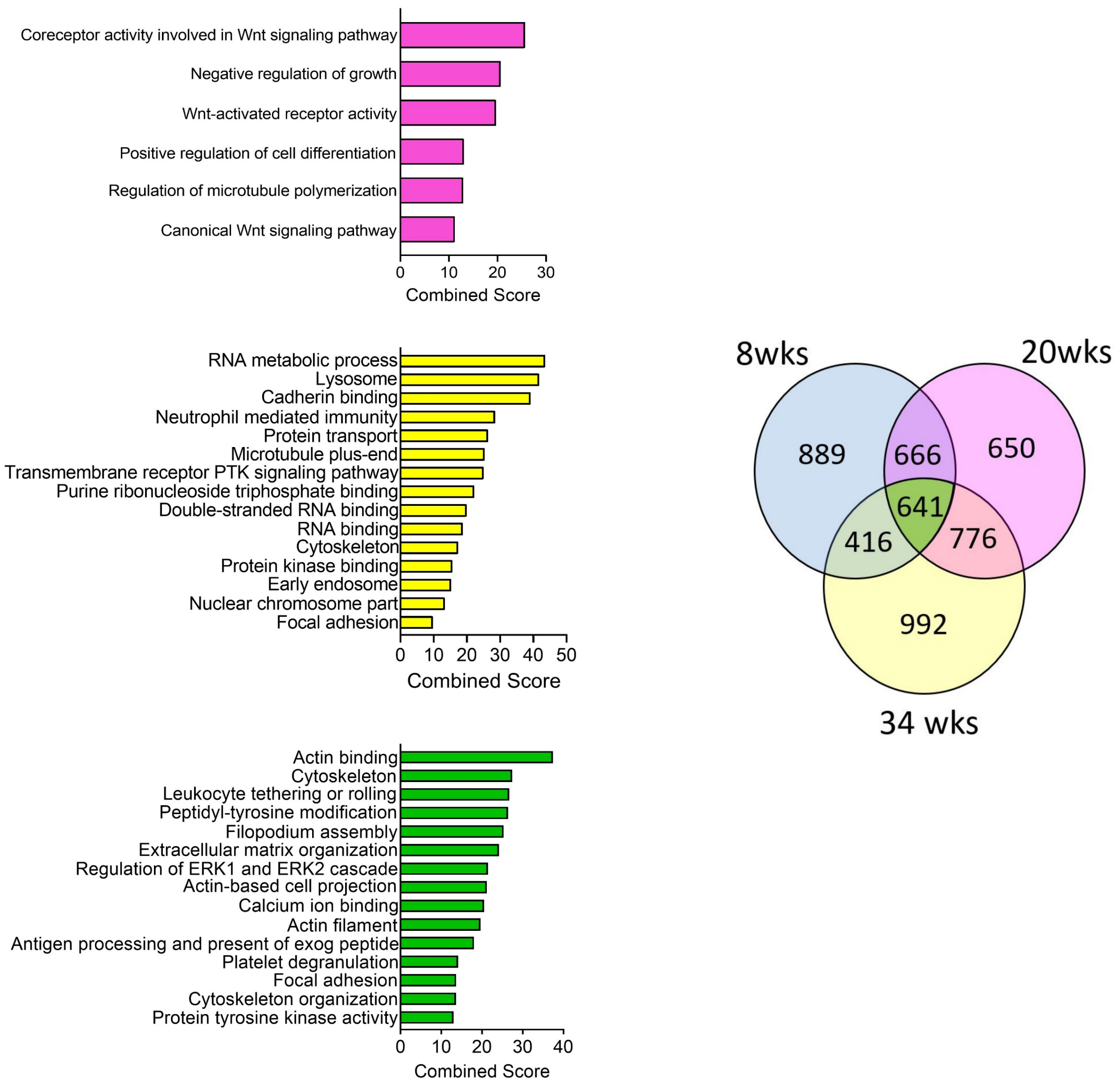

Supplemental Figure 3: GO term pathway analysis of upregulated genes in isolated adipocytes.

Genes were deemed significant with padj<0.05. GO term pathway analysis was done using EnrichR and Revigo softwares [32-34]. Combined scores were used to rank the GO terms and the top 15 or less are represented here.

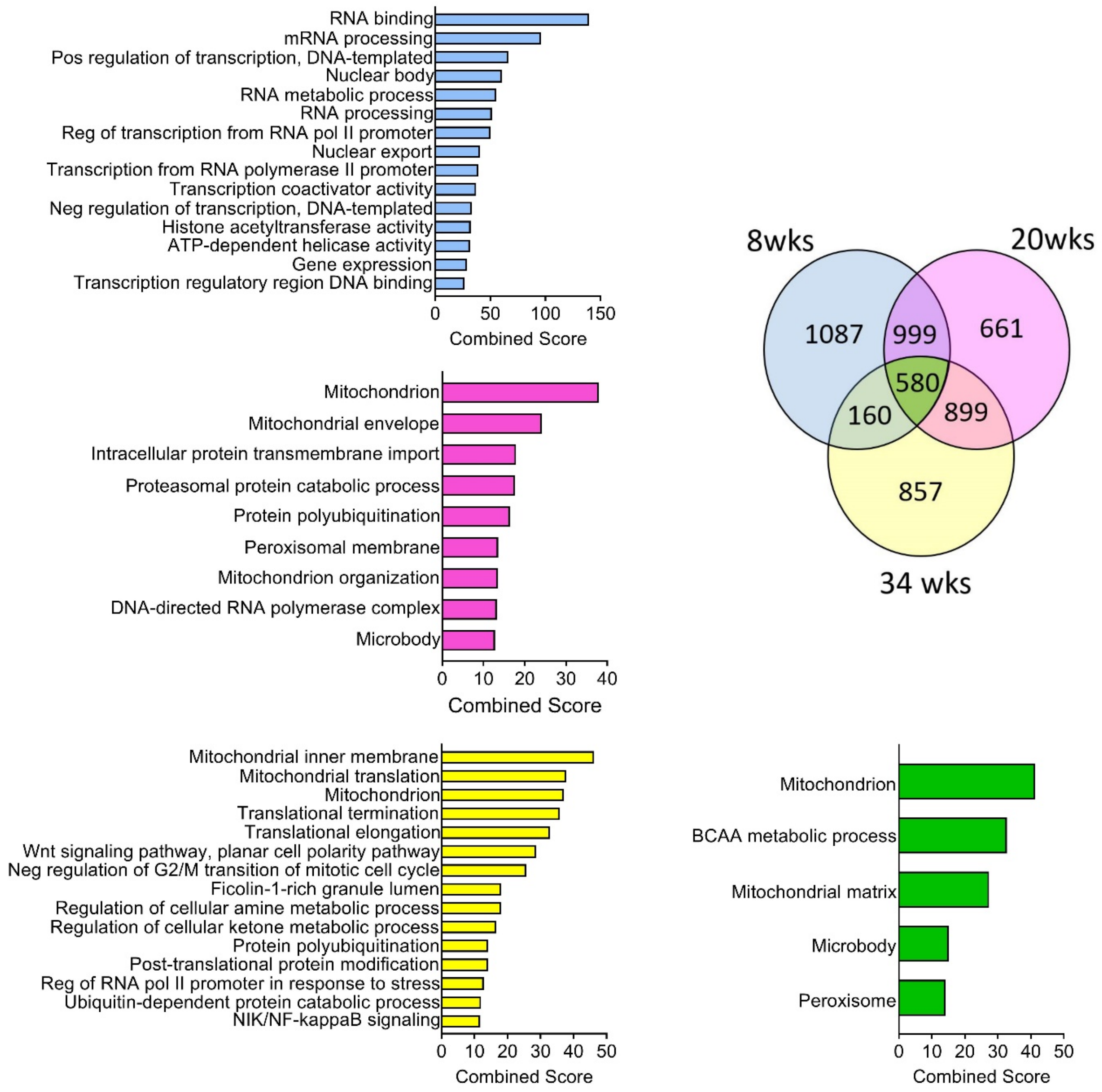

Supplemental Figure 4: GO term pathway analysis of downregulated genes in isolated adipocytes.

Genes were deemed significant with padj<0.05. GO term pathway analysis was done using EnrichR and Revigo softwares [32-34]. Combined scores were used to rank the GO terms and the top 15 or less are represented here.

HSL, ATGL (Figure 5C)

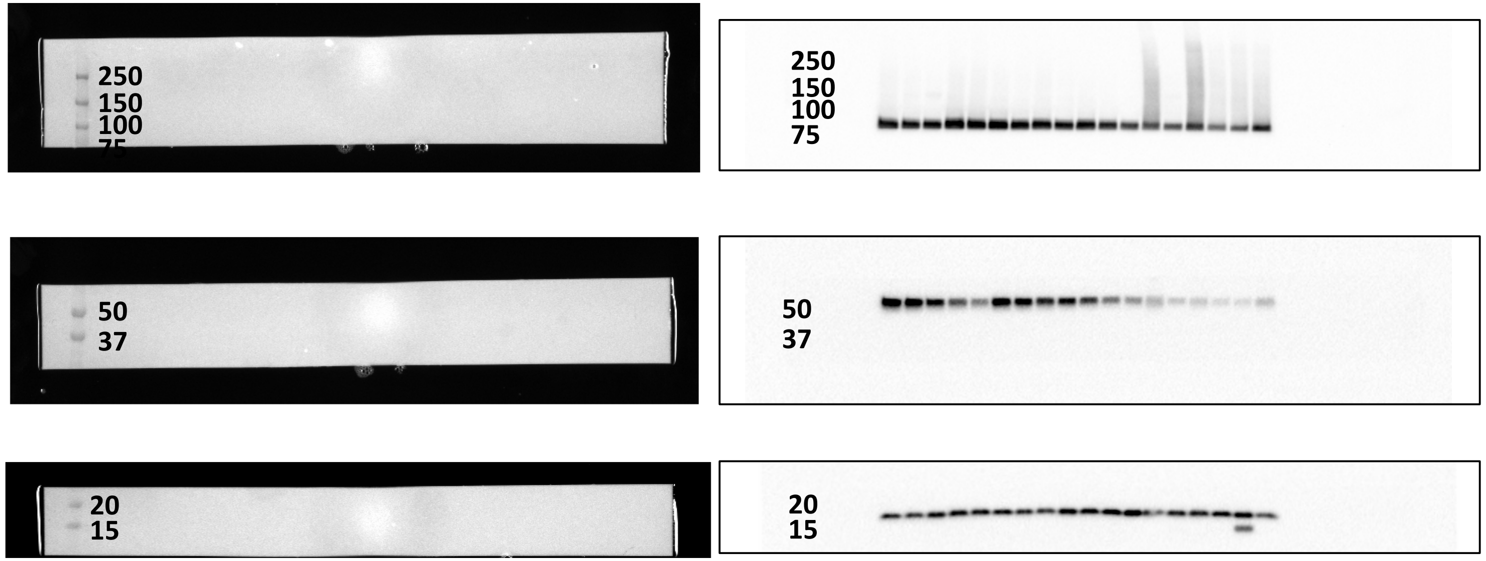

HSL (~81,83 kDa); top section of blot

ATGL (~54 kDa); middle section of blot

Cyclophilin A (~18 kDa); bottom section of blot)

Molecular weight ladder:
Biorad Precision Plus Protein Dual Color Standards #1610374

℗-HSL ser563, MAGL (Figure 5C)

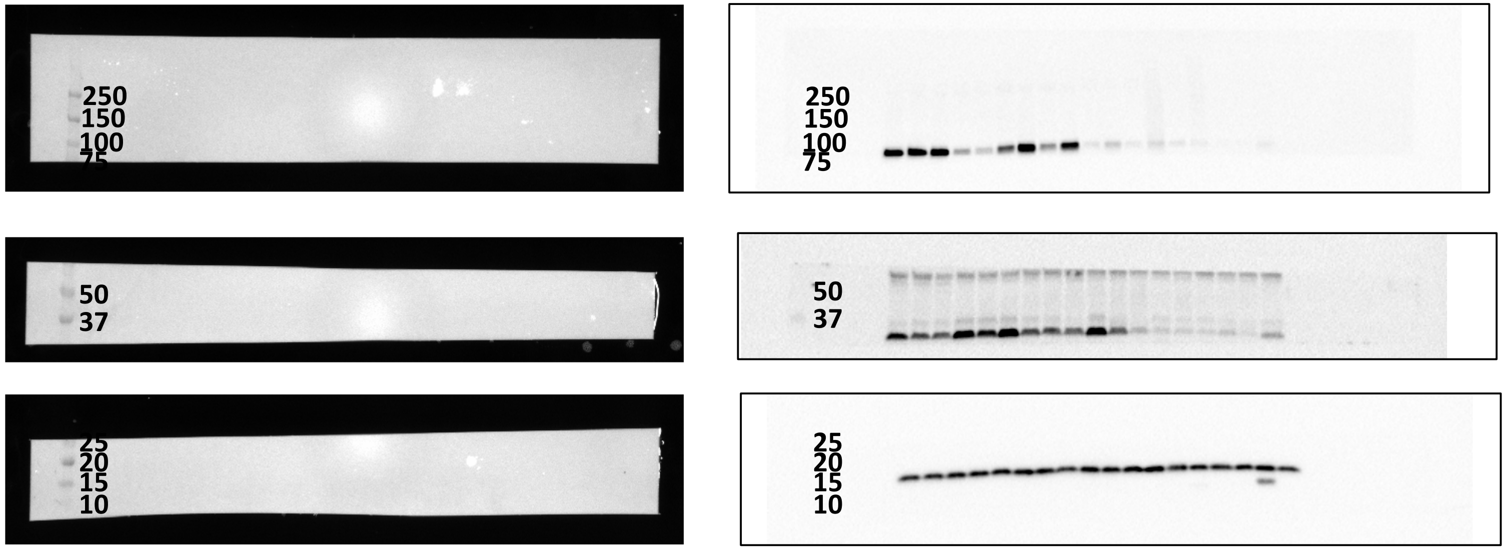

℗-HSLser563 (~81,83 kDa); top section of blot)

MAGL (~35 kDa); middle section of blot

Cyclophilin A (~18 kDa); bottom section of blot

Molecular weight ladder:
Biorad Precision Plus Protein Dual Color Standards #1610374

Adiponectin (Figure 5D)

White light image of blot

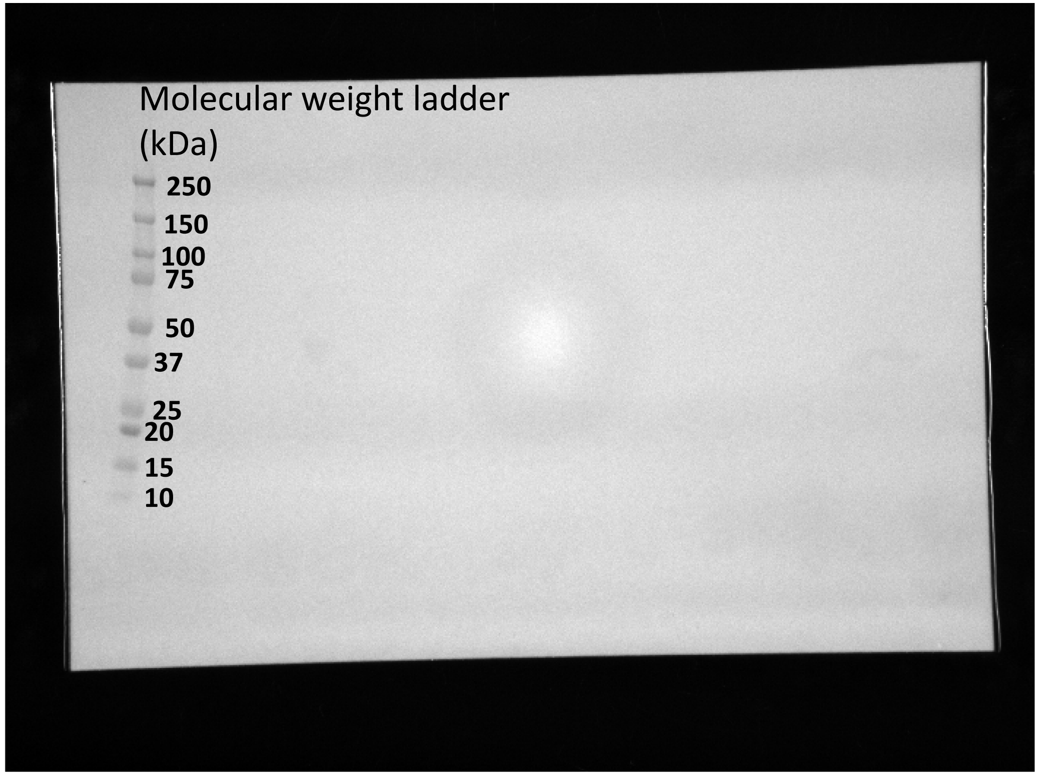

Chemiluminescent image of blot

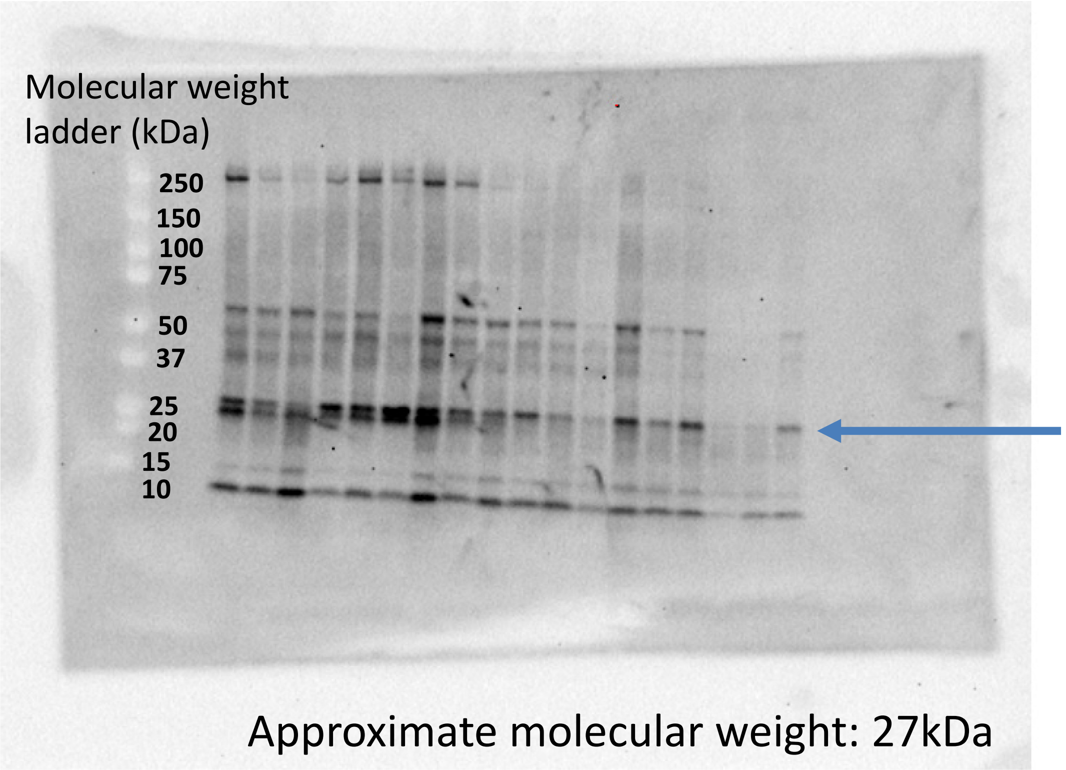

Molecular weight ladder:
Biorad Precision Plus Protein Dual Color Standards #1610374

Cyclophilin A (Figure 5D)

White light image of blot

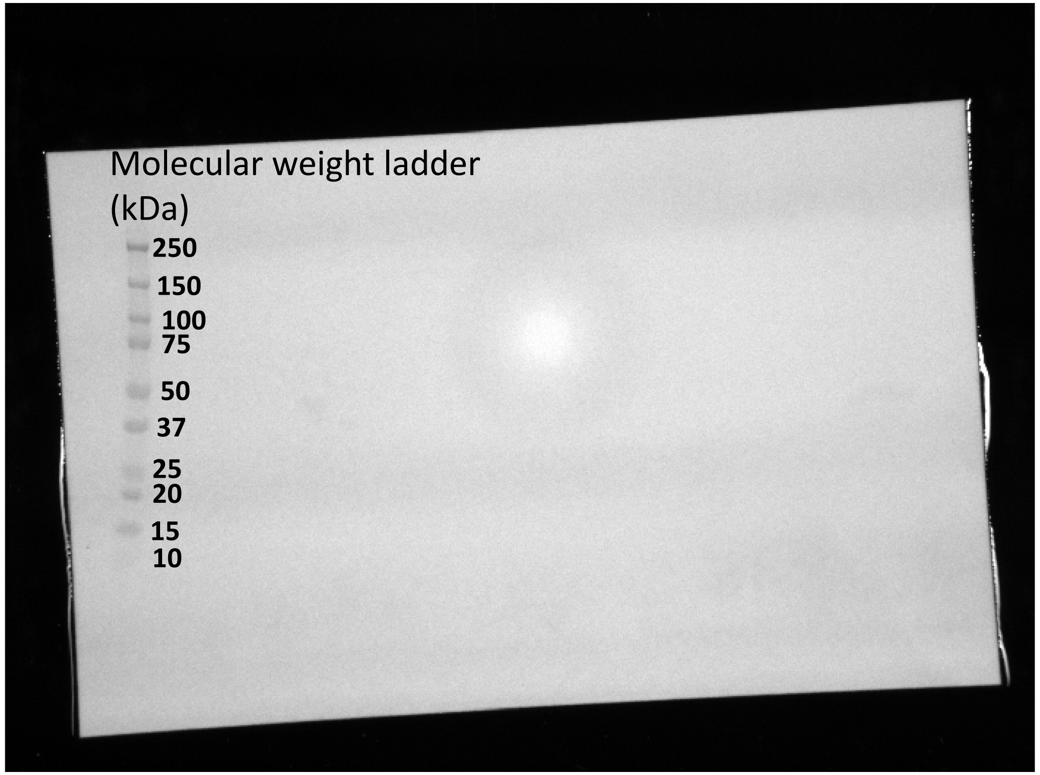

Chemiluminescent image of blot

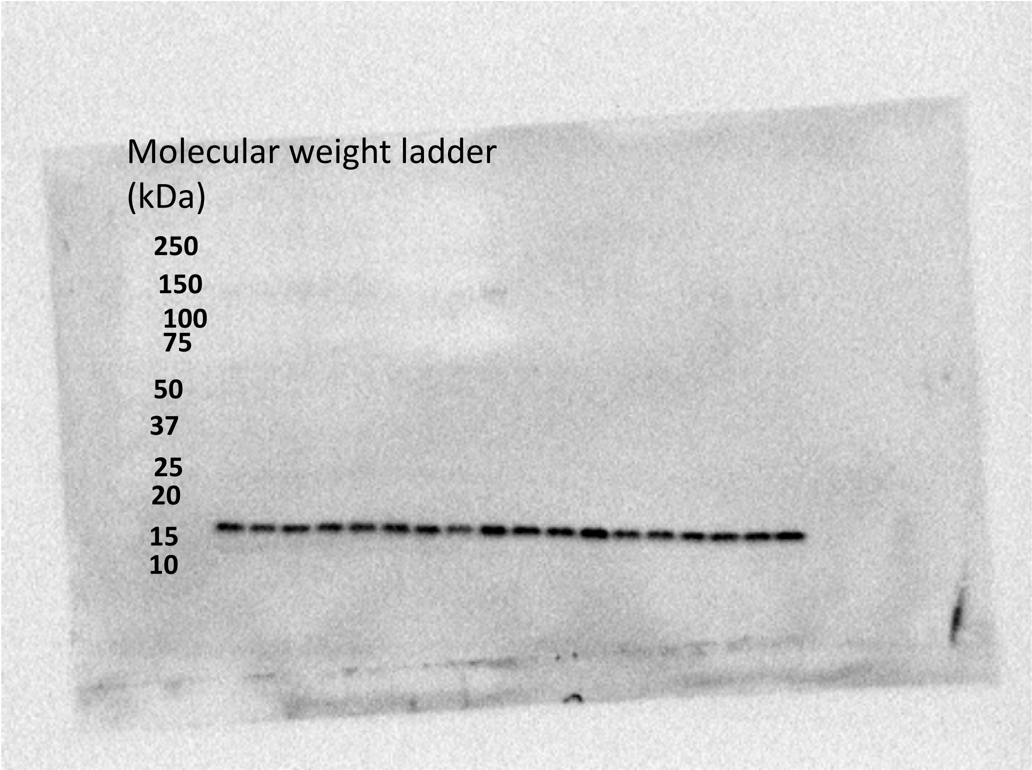

Molecular weight ladder:
Biorad Precision Plus Protein Dual Color Standards #1610374

Approximate molecular weight: 18kDa

Supplemental Figure 5: Original blots from which Figures 5C and 5D were constructed.
